# An integrated omics analysis reveals molecular mechanisms that are associated with differences in seed oil content between *Glycine max* and *Brassica napus*

**DOI:** 10.1186/s12870-018-1542-8

**Published:** 2018-12-04

**Authors:** Zhibin Zhang, Jim M. Dunwell, Yuan-Ming Zhang

**Affiliations:** 1 Crop Information Center, College of Plant Science and Technology, Huazhong Agricultural University, Wuhan, 430070 China; 2 Zhengzhou Research Base, State Key Laboratory of Cotton Biology, Zhengzhou University, Zhengzhou, 450000 China; 3 School of Agriculture, Policy and Development, University of Reading, Reading, RG6 6AS UK

**Keywords:** *Glycine max*, *Brassica napus*, Acyl-lipid biosynthesis, Transcription factor, miRNA, Gene network

## Abstract

**Background:**

Rapeseed (*Brassica napus* L.) and soybean (*Glycine max* L.) seeds are rich in both protein and oil, which are major sources of biofuels and nutrition. Although the difference in seed oil content between soybean (~ 20%) and rapeseed (~ 40%) exists, little is known about its underlying molecular mechanism.

**Results:**

An integrated omics analysis was performed in soybean, rapeseed, *Arabidopsis* (*Arabidopsis thaliana* L. Heynh), and sesame (*Sesamum indicum* L.), based on *Arabidopsis* acyl-lipid metabolism- and carbon metabolism-related genes. As a result, candidate genes and their transcription factors and microRNAs, along with phylogenetic analysis and co-expression network analysis of the *PEPC* gene family, were found to be largely associated with the difference between the two species. First, three soybean genes (*Glyma.13G148600, Glyma.13G207900* and *Glyma.12G122900*) co-expressed with *GmPEPC1* are specifically enriched during seed storage protein accumulation stages, while the expression of *BnPEPC1* is putatively inhibited by bna-miR169, and two genes *BnSTKA* and *BnCKII* are co-expressed with *BnPEPC1* and are specifically associated with plant circadian rhythm, which are related to seed oil biosynthesis. Then, in de novo fatty acid synthesis there are rapeseed-specific genes encoding subunits β-CT (*BnaC05g37990D*) and BCCP1 (*BnaA03g06000D*) of heterogeneous ACCase, which could interfere with synthesis rate, and *β-CT* is positively regulated by four transcription factors (*BnaA01g37250D, BnaA02g26190D, BnaC01g01040D* and *BnaC07g21470D*). In triglyceride synthesis, *GmLPAAT2* is putatively inhibited by three miRNAs (gma-miR171, gma-miR1516 and gma-miR5775). Finally, in rapeseed there was evidence for the expansion of gene families, CALO, OBO and STERO, related to lipid storage, and the contraction of gene families, LOX, LAH and HSI2, related to oil degradation.

**Conclusions:**

The molecular mechanisms associated with differences in seed oil content provide the basis for future breeding efforts to improve seed oil content.

**Electronic supplementary material:**

The online version of this article (10.1186/s12870-018-1542-8) contains supplementary material, which is available to authorized users.

## Background

Seed storage lipids not only provide food for human dietary consumption, but are also increasingly used as renewable sources for biofuels [1, 2]. In oil crops, such as *Arabidopsis*, soybean, rapeseed and sesame, seed oil content varies from 20 to 60%. Interestingly, the total seed storage reserves in soybean seed, consisting of ~ 20% oil and ~ 40% protein [3], is almost equal to the protein (~ 20%) and oil (~ 40%) contents in rapeseed [4]. As we know, most of the raw material required for seed oil and protein biosynthesis in rapeseed and soybean are derived from carbohydrate degradation [5]. And it should be noted that substrate competition between seed oil and protein synthesis exists in oilseed crops [6, 7]. This is because phosphoenolpyruvate (PEP), a carbon compound derived from glycolysis, is not only used to synthesize acetyl-Coenzyme A (acetyl-CoA), which serves as a substrate in the first step of de novo fatty acid synthesis, but is also required for the synthesis of oxaloacetate (OAA), which serves as a substrate in amino acid synthesis. Thus, carbon metabolism is related to oil synthesis, and boosting the carbon flow to lipid synthesis can significantly increase seed oil content [8].

In the past several decades, more than 700 acyl-lipid metabolism-related genes and several hundred genes participating in carbohydrate metabolism have been identified in *Arabidopsis thaliana* [9, 10]. Among these genes, more than 280 have been confirmed in *A. thaliana* mutants as associated with acyl-lipid metabolism (http://aralip.plantbiology.msu.edu) [11]. Meanwhile, many genes have been experimentally validated to be closely related to seed oil content. For example, phosphoenolpyruvate carboxylase (*PEPC*) in cotton [12], acetyl-CoA carboxylase (*ACCase*) in rapeseed [13] and potato [14] participate in de novo fatty acid biosynthesis; fatty acylthioesterase B (*GmFatB*) in soybean [15] and patatin-related phospholipase As (*pPLAs*) in *Arabidopsis* [16] are involved in fatty acid elongation; glycerol-3-phosphate dehydrogenase (*GPDH*) in rapeseed [17], glycerol-3-phosphate acyltransferase (*GPAT*) in *Arabidopsis* [18], 2-lysophosphatidic acid acyltransferase (*LPAAT*) in *Arabidopsis* [19] and cotton [20], acyl-CoA: diacylglycerol acyltransferase (*DGAT*) in *Arabidopsis* [21, 22], maize [23], and rapeseed [24] are related to TAG synthesis; oleosins (*OLE1)* in *Arabidopsis* [25] participates in lipid droplet assembly and storage. In addition, some transcription factors (TFs) have been found to be associated with seed oil content, i.e., WRINKLED1 (WRI1) [26], LEAFY COTYLEDON1 (LEC1) [27, 28], LEAFY COTYLEDON2 (LEC2) [29], FUSCA3 (FUS3) [30], GmDof4 and GmDof11 [31], GmbZIP123 [32], GmMYB73 [33], GmDREBL [34], GmNFYA [35], GmZF351 [36], and ABSCISIC ACID INSENSITIVE3 (ABI3) [37, 38]. However, all the above studies involved only a single lipid-related gene or transcription factor. Seed oil content is typically a quantitative trait regulated by multiple genes. As we know, these genes have been identified in the form of quantitative trait loci in soybean and rapeseed in the past decades [39–43].

In reality, acyl-lipid metabolism is a complex biological process that includes at least conversion of sucrose to pyruvate, plastidial de novo fatty acid (FA) synthesis, endoplasmic triacylglycerol (TAG) biosynthesis, and oil-body assembly. It is therefore important to determine whether specific combination of multiple genes from multiple metabolic pathways can increase seed oil content more effectively as compared with the manipulation of an individual gene. For example, it was found that *Arabidopsis* seed-specific overexpression of *WRI1* and *DGAT1* combined with suppression of *SDP1* leads to higher seed oil content than the manipulation of each gene individually [44]. Additionally, the simultaneous overexpression of *GmFabG* (*Glyma.12G092900*), *GmACP* (*Glyma.09G060900*) and *GmFAD8* (*Glyma.03G056700*) can significantly increase soybean seed oil content [45]. The down-regulation of *CaFAD2* and *CaFAE1* in crambe with the *FAD2-FAE1* RNAi vector led to a significant increase in the seed oil to 80% compared to 13% for the wild type [46]. Seed-specific simultaneous overexpression of *BnGPDH, BnGPAT* and *ScLPAAT* genes in transgenic rapeseed may further enhance the desirable oil content relative to single-gene overexpression [47]. Moreover, Yu et al. [48] developed a complete analysis platform of functional annotation for the soybean genes involved in acyl-lipid metabolism, and this makes the study of acyl lipid metabolism more efficient and accurate. However, none of the above studies were conducted at the whole genome level.

With the rapid development of sequencing technology, more and more plant genomes have been sequenced, and this accelerates the progress of research on acyl-lipid metabolism [49–53]. Troncoso-Ponce et al. [10] showed that the expression stoichiometry of most key lipid-related genes was relatively conserved during seed development in *Ricinus communis*, *Brassica napus*, *Euonymus alatus* and *Tropaeolum majus*. Wang et al. [54] analyzed the expression differences of key ovule-specific genes between non-fibrous Raymond’s cotton and the upland cotton with fiber. Zhang et al. [55] dissected the molecular mechanisms of differences in seed oil content between four high-oil dicotyledons and three low-oil grass plants. However, little is known about the molecular mechanism for the difference of seed oil contents between soybean and rapeseed.

To understand the molecular mechanisms of the difference of seed oil content between rapeseed and soybean, an integrated omics analysis was performed in *Arabidopsis*, rapeseed, soybean and sesame based on *Arabidopsis* acyl-lipid metabolism- and carbon metabolism-related genes. The integrated omics analysis included gene copy number variation, expression pattern, microRNA/transcription factor, phylogenetic and co-expressional network analyses. Thus, candidate genes, transcription factors, and microRNAs that may be responsible for the difference were identified. These results provide a novel explanation for differences at the whole genome level and the basis for future breeding efforts to improve seed oil content in oilseed crops.

## Results

### Identification of candidate genes related to lipid biosynthesis

To identify orthologous genes in soybean and rapeseed, we used OrthoMCL to cluster putative OGs of genes across *Arabidopsis,* soybean*,* rapeseed and sesame. As a consequence, 172,626 (81.83%) protein-coding genes from the four species were clustered into 27,236 OGs (Additional file 1: Table S1), with each group representing a gene family. Among these gene families, 11,314 (41.54%) were defined as rapeseed-specific paralogous gene clusters without soybean genes, and only 3391 (12.45%) were soybean-specific families (Additional file 1: Table S2). In addition, 1687 OGs were identified to have one copy of a soybean gene and multiple copies for rapeseed genes, and 446 OGs with one copy for the rapeseed gene and multiple copies for soybean genes (Additional file 1: Table S2).

To date, more than 700 *Arabidopsis* acyl-lipid metabolism genes have been detected, of which 135 are directly involved in the processes of de novo fatty acid synthesis, triglyceride biosynthesis and lipid droplet formation. Using the method as described by Troncoso-Ponce et al. [10], we also extracted 238 *Arabidopsis* carbon-metabolism genes. Subsequently, searches using the 373 genes as queries were performed to obtain lipid biosynthesis-related homologous gene families (Additional file 1: Table S3). As a result, 230 putative OGs related to lipid synthesis were identified, which include 781 soybean genes and 1267 rapeseed genes (Additional file 1: Table S4).

### Copy number variation and expression clustering of candidate genes related to lipid biosynthesis

In order to eliminate the effect of species ploidy, the relative copy number of a gene was used to measure the difference of copy number of homologous genes between species. Of the above 230 OGs related to lipid synthesis, 44 were found to have differences in relative copy number of a gene between soybean and rapeseed (Additional file 1: Table S5).

To cluster and visualize the expressional patterns of all 2048 rapeseed and soybean genes in the 230 OGs, we exploited STEM software [56] to analyze the expression data of four seed development stages in rapeseed (GSE77637, [57]) and soybean (GSE42871, [58]). In this study, R3, R4, R7 and R8 stages in soybean and 2, 4, 6, and 8 weeks after pollination (WAP) in rapeseed were defined as t1’, t2’, t3’ and t4’ in soybean and t1, t2, t3 and t4 in rapeseed, respectively. Results showed that 2048 genes were grouped into 20 clusters, including three up-regulation patterns (cluster13, cluster16 and cluster18) and one down-regulation profile (cluster3) during stages with rapid accumulation of seed oil (Additional file 2: Figure S1). Additionally, there were 23, 202, 86 and 22 genes in cluster3, cluster13, cluster16 and cluster18, respectively (*P*-value < 0.05) (Additional file 2: Figure S2).

**Fig. 1:**
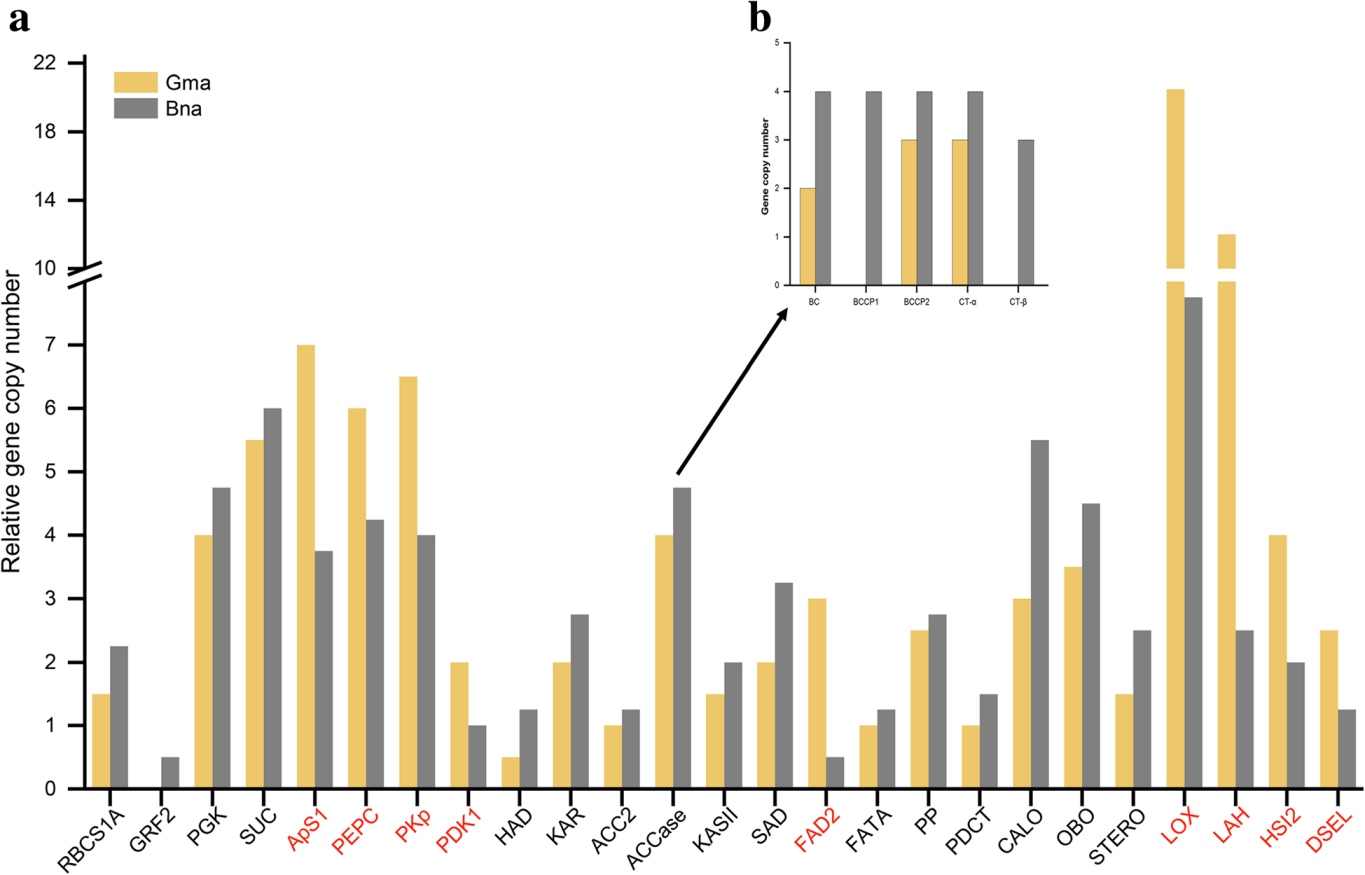
Candidate genes for the difference of seed oil content between soybean and rapeseed. **a** Relative copy number variation analysis of candidate genes contributed to the difference of seed oil contents between soybean and rapeseed. The red genes in X-axis indicate more relative gene copies in rapeseed over in soybean, and the opposite situation is expressed in black. **b** PK in plastid is composed of Alpha (α) and Beta (β) subunits. ACCase contains homogeneous structure ACC2 and heterogeneous ACCase complex including α-CT, β-CT, BC, BCCP1 and BCCP2 subunits

Based on the above two results, 192 soybean and 292 rapeseed candidate genes were inferred to be related to the differences of seed oil content between rapeseed and soybean. According to the sequence homology with *Arabidopsis* genes, 484 genes were found to putatively encode a series of core enzymes. For example, GRF2 and RBCS1A during photosynthesis; PGK, ApS1, SUC, PEPC and PKp involved in carbon metabolism from sucrose to pyruvate; PDK1, ACCase, KASII, HAD, KAR, FATA, SAD and FAD2 in de novo fatty acid biosynthesis; and PAP, PDCT participating in TAG synthesis, as well as oil-body proteins OBO, CALO, STERO, and oil degradation genes LOX, LAH, HSI2 and DSEL (Fig. 1).

To further determine the functions of the above 484 genes, KEGG enrichment analysis was conducted using KOBAS 2.0 [59]. As a result, the top 10 KEGG pathways for soybean and rapeseed candidate genes were obtained (Table 1). It was found that the two crops had eight KEGG pathways in common, namely pyruvate metabolism, carbon metabolism, biosynthesis of secondary metabolites, glycolysis, purine metabolism, biosynthesis of amino acids, carbon fixation in photosynthetic organisms, and glycerophospholipid metabolism. In addition, fatty acid biosynthesis in soybean is similar to fatty acid metabolism in rapeseed. The results are consistent with those in Troncoso-Ponce et al. [10] and Ohlrogge and Browse [60], and thus ensure the reliability of candidate genes in the next analysis.

**Table 1:** KEGG pathway enrichment analysis for candidate genes related to the differences of seed oil content between rapeseed and soybean

KEGG pathways	Soybean					Rapeseed				
	ID	Input number	Background number	*P*-value	Corrected *P*-value	ID	Input number	Background number	*P*-value	Corrected *P*-value
Pyruvate metabolism	ath00620	43	85	4.92E-80	3.65E-77	ath00620	80	85	1.03E-133	1.64E-132
Carbon metabolism	ath01200	41	262	6.29E-58	2.34E-55	ath01200	75	262	2.99E-94	2.39E-93
Biosynthesis of secondary metabolites	ath01110	42	1076	1.04E-35	3.52E-34	ath01110	85	1076	3.90E-65	1.25E-64
Glycolysis	ath00010	21	117	3.37E-30	4.24E-29	ath00010	38	117	1.89E-48	5.03E-48
Purine metabolism	ath00230	21	158	1.06E-27	9.51E-27	ath00230	38	158	3.43E-44	7.84E-44
Biosynthesis of amino acids	ath01230	21	255	1.18E-23	7.77E-23	ath01230	38	255	3.31E-37	5.29E-37
Carbon fixation in photosynthetic organisms	ath00710	12	69	1.97E-17	9.34E-17	ath00710	17	69	2.36E-20	2.90E-20
Glycerophospholipid metabolism	ath00564	15	86	1.43E-21	8.06E-21	ath00564	30	86	4.48E-39	7.97E-39
Fatty acid biosynthesis	ath00061	17	41	6.76E-30	8.37E-29					
Fatty acid metabolism						ath01212	46	67	8.95E-71	3.58E-70
Glycerolipid metabolism	ath00561	11	53	8.03e-17	3.53e-16					
Biotin metabolism						ath00780	8	14	1.32E-12	1.40E-12

### Expression profiles of candidate genes responsible for the difference of seed oil content between rapeseed and soybean

The transcriptomic datasets from the seed developmental stages in soybean (GSE42871) and rapeseed (GSE77637) downloaded from the GEO (Gene Expression Omnibus) database were used to validate the above candidate genes. To compare the expressional profiles of each candidate gene in soybean and rapeseed, relative expression content for each gene was adopted in this study; this is defined as the ratio of the expression of each gene to average expression of all the genes in the species. As a result, a majority of candidate genes in rapeseed, except for phosphoenolpyruvate carboxylase (PEPC), Ribulose-1,5-bisphosphate carboxylase/oxygenase small subunit (RBCS1A), lipoxygenase (LOX) and steroleosin (STERO), had higher relative expression than those in soybean, especially for PK and ACCase (Additional file 2: Figure S3, Additional file 1: Table S6).

More importantly, we noted some interesting phenomena. First, *GmPEPC* had higher relative expression at the early and middle seed development stages than *BnPEPC* (Fig. 3), indicating that PEP may be more likely to be used to synthesize protein in soybean seed, because PEPC, a member of carboxyl lyase family, catalyzes phosphoenolpyruvate (PEP) to produce oxaloacetic acid (OAA) for amino acid biosynthesis. Then, the relative expression contents of rapeseed genes encoding four subunits (α-CT, β-CT, BC and BCCP) of heterogeneous acetyl-CoA carboxylase (ACCase), catalyzing the first and committed reaction of de novo fatty acid biosynthesis in plastids, were higher than those of soybean genes in the oil rapid accumulation stages. Especially, BCCP1 and β-CT were not expressed during soybean seed development (Additional file 2: Figure S4). Finally, *GmPEPC1* (*Glyma.06G277500*) and *GmPEPC3* (*Glyma.06G229900*) had higher relative expression than PKp and ACCase in soybean. Conversely, *PKp-β* (*BnaC02g44850D*), *PKp-α* (*BnaA01g24280D*) and ACCase had higher relative expression than *PEPC* in rapeseed development stages (Additional file 2: Figure S4). Therefore, we deduced that *PEPC*, *PKp* and *ACCase* are most likely to be the key genes that regulate the distribution of carbon sources in soybean and rapeseed seeds.

### Transcription factors and microRNAs regulatory network analysis of the candidate genes

To clarify the differences of regulatory networks of key candidate genes in soybean and rapeseed, we identified transcription factors (TFs) related to lipid biosynthesis in seed development. TFs and their target genes were downloaded from PlantTFDB v3.0 [61]. Together with the results mentioned above, it was found that the rapeseed-specific gene (*BnaA10g13960D*), encoding β-CT subunit of ACCase, is positively regulated by four zinc finger TFs, namely *BnaA01g37250D, BnaA02g26190D, BnaC01g01040D and BnaC07g21470D* (Table 2; Additional file 2: Figure S5) [62].

**Table 2:** The microRNAs regulating rapeseed *PEPC* gene (*BnaA03g33640D*) and soybean *LPAAT2* gene (*Glyma.03G139700*)

miRNA_Acc.	Target_Acc.	Expectation	UPE	miRNA_start	miRNA_end	Target_start	Target_end	miRNA_aligned_fragment	Target_aligned_fragment	Inhibition
bna-miR169a	*BnaA03g33640D*	4	21.988	1	20	204	223	CAGCCAAGGAUGACUUGCCG	AGGCAAGCCAAACUUGGCUG	Translation
bna-miR169b	*BnaA03g33640D*	4	21.988	1	20	204	223	CAGCCAAGGAUGACUUGCCG	AGGCAAGCCAAACUUGGCUG	Translation
bna-miR169c	*BnaA03g33640D*	3.5	21.988	1	20	204	223	UAGCCAAGGAUGACUUGCCU	AGGCAAGCCAAACUUGGCUG	Translation
bna-miR169d	*BnaA03g33640D*	3.5	21.988	1	20	204	223	UAGCCAAGGAUGACUUGCCU	AGGCAAGCCAAACUUGGCUG	Translation
bna-miR169e	*BnaA03g33640D*	3.5	21.988	1	20	204	223	UAGCCAAGGAUGACUUGCCU	AGGCAAGCCAAACUUGGCUG	Translation
bna-miR169f	*BnaA03g33640D*	3.5	21.988	1	20	204	223	UAGCCAAGGAUGACUUGCCU	AGGCAAGCCAAACUUGGCUG	Translation
bna-miR169g	*BnaA03g33640D*	3.5	21.988	1	22	202	223	UAGCCAAGGAUGACUUGCCUGC	GAAGGCAAGCCAAACUUGGCUG	Translation
bna-miR169h	*BnaA03g33640D*	3.5	21.988	1	22	202	223	UAGCCAAGGAUGACUUGCCUGC	GAAGGCAAGCCAAACUUGGCUG	Translation
bna-miR169i	*BnaA03g33640D*	3.5	21.988	1	22	202	223	UAGCCAAGGAUGACUUGCCUGC	GAAGGCAAGCCAAACUUGGCUG	Translation
bna-miR169j	*BnaA03g33640D*	3.5	21.988	1	22	202	223	UAGCCAAGGAUGACUUGCCUGC	GAAGGCAAGCCAAACUUGGCUG	Translation
bna-miR169k	*BnaA03g33640D*	3.5	21.988	1	22	202	223	UAGCCAAGGAUGACUUGCCUGC	GAAGGCAAGCCAAACUUGGCUG	Translation
bna-miR169l	*BnaA03g33640D*	3.5	21.988	1	22	202	223	UAGCCAAGGAUGACUUGCCUGC	GAAGGCAAGCCAAACUUGGCUG	Translation
bna-miR169n	*BnaA03g33640D*	4	21.988	1	20	204	223	CAGCCAAGGAUGACUUGCCG	AGGCAAGCCAAACUUGGCUG	Translation
gma-miR171b-3p	*Glyma.03G139700*	3	24.213	1	20	168	186	CGAGCCGAAUCAAUAUCACU	AGUGAUAUUGAUU-GGCUUG	Cleavage
gma-miR1516a-5p	*Glyma.03G139700*	3	24.131	1	23	283	305	CAAGUUAUAAGCUCUUUUGAGAG	CUCUCAAAAGCACUUAUGGCUUG	Cleavage
gma-miR1516b	*Glyma.03G139700*	1	14.793	1	21	264	284	AGCUUCUCUACAGAAAAUAUA	UAUAUUUUCAGUAGAGAAGCU	Cleavage
gma-miR5775	*Glyma.03G139700*	3	23.458	1	21	279	299	AUAAGCUCUUUUGAGAGCUUC	GAAGCUCUCAAAAGCACUUAU	Cleavage
gma-miR171b-3p	*Glyma.19G142500*	3	22.553	1	20	210	228	CGAGCCGAAUCAAUAUCACU	AGUGAUAUUGAUU-GGCUUG	Cleavage

Meanwhile, 639 soybean and 92 rapeseed mature microRNAs were downloaded from miRBase (version 21). Their target genes were predicted using psRNATarget, a plant small RNA target analysis server. As a result, 4411 soybean and 1780 rapeseed miRNA-Target gene pairs were obtained. Among these genes, 116 and 61 were associated with lipid synthesis in soybean and rapeseed, respectively. Note that bna-miR169 inhibits the expression of the *BnPEPC* gene; this may facilitate a greater carbon flow to de novo fatty acid synthesis, and the expression of *GmLPAAT2* gene is putatively inhibited by gma-miR171, gma-miR1516 and gma-miR5775 (Table 2; Additional file 2: Figure S5).

### Evolutionary analysis of PEPC gene family

#### Phylogenetic analysis and conserved motifs analysis of PEPC gene family

**Fig. 2:**
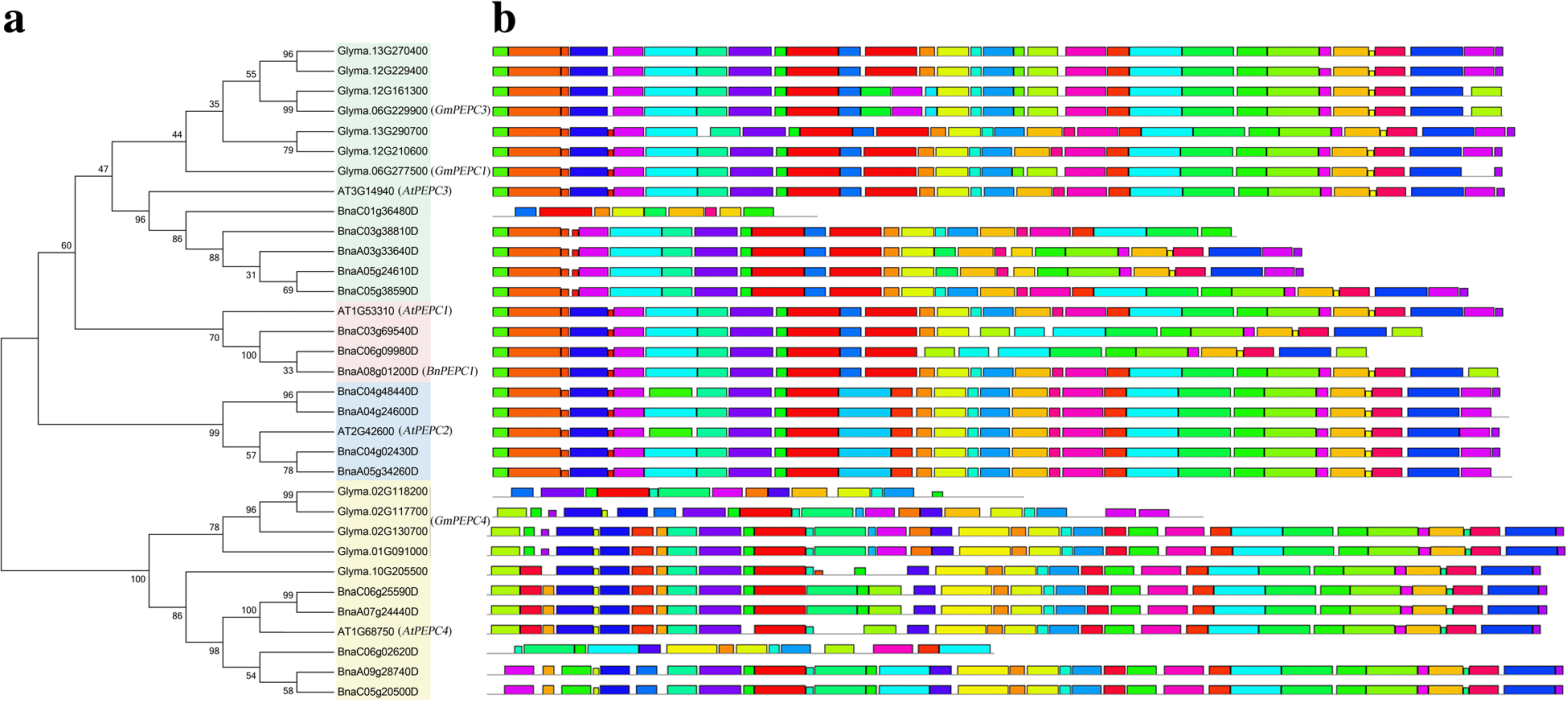
Phylogenetic tree and gene motif analysis of PEPC gene family. **a** Phylogenetic tree of PEPC gene family is constructed from the complete alignment of 33 PEPC protein sequences in *Arabidopsis*, soybean, and rapeseed using the neighbor-joining method with 1000 bootstrap replicates with the MEGA 7.0 software program. The bootstrap scores are indicated on the nodes, and the 4 PEPC branches, all of which are based on *Arabidopsis* PEPC orthologous genes, are indicated in four color boxes. The representative gene of each branch is shown followed by an additional abbreviation. **b** Conserved domains analysis of PEPCs. The domains of soybean genes in *AtPEPC3* branch are relatively more conservative compared with rapeseed genes. And the domains of rapeseed genes in *AtPEPC1* and *AtPEPC2* branches are relatively conservative. However, the differences of gene domain in the bacterial *AtPEPC4* branch are significant between soybean and rapeseed

Three plant-type PEPC genes (PTPCs) (*AtPEPC1*, *AtPEPC2* and *AtPEPC3*) and one bacterial-type PEPC genes (BTPCs) *(AtPEPC4)* exist in *A. thaliana* [63]. To investigate the evolution of the PEPC gene family in soybean and rapeseed, the full amino acid sequences encoded by 33 PEPC genes in soybean, rapeseed and *Arabidopsis* were used to construct a phylogenetic tree using a neighbor-joining method. As a result, the PEPC genes were grouped into two distinct families with four subfamilies, which are consistent with those in *A. thaliana* (Fig. 2). In Fig. 2, *GmPEPC1* is close to *GmPEPC3* in its evolutionary relationship, and their expression patterns during seed development in Additional file 2: Figure S4 are complementary. Meanwhile, the conserved motifs of PEPC genes were further analyzed using MEME [64]. Results showed that the motif structure of *GmPEPC3* was more conserved than that of *BnPEPC3*, and the motif structures of *BnPEPC1* and *BnPEPC2* were also relatively more conserved than that of *BnPEPC3* (Fig. 2). Note that there are distinct differences of BTPCs between soybean and rapeseed, indicating the existence of its extensive functional differentiation.

#### Evolutionary rate and positive selection analysis of PEPC genes

To determine whether the genes of the PEPC gene family are under different evolutionary constraints in soybean and rapeseed, the ω (Ka/Ks) values for the above genes were calculated using the branch model (BM) and the branch-site model (BSM) of the Codeml program in PAML. As a result, the evolution rate ω_0_ (Ka/Ks) was estimated to be 0.340 and log-likelihood was − 2343.655 if the evolution rates at all branches were the same; six evolution rates (ω) were estimated to be 0.078, 0.054, 0.106, 0.093, 0.495 and 0.175 and log-likelihood was − 2361.163 if the evolution rates changed across different branches (Additional file 3: Table S7; Additional file 2: Figure S6). Clearly, there was a significant difference between the above two models (Additional file 3: Table S7, *P*-value = 4.278e-06). We also found that the ω value for the bacterial-type PEPC soybean sub-branch (ω = 0.495) was significantly higher than the ω_0_ value (= 0.340) and the ω values for the other sub-branches were significantly lower than the ω_0_ value (= 0.340). This indicates that the BTPC genes have experienced positive selection and the PTPC genes have experienced purifying selection. Furthermore, the BSM model was used to identify positively selected sites. As a result, we observed significant positive selection for soybean bacterial-type PEPC genes and the amino acid sites of positive selection, associated with *GmPEPC4*, were 56F and 61 V (Additional file 3: Table S8).

### Differential analysis of PEPC1 gene co-expression networks between soybean and rapeseed

**Fig. 3:**
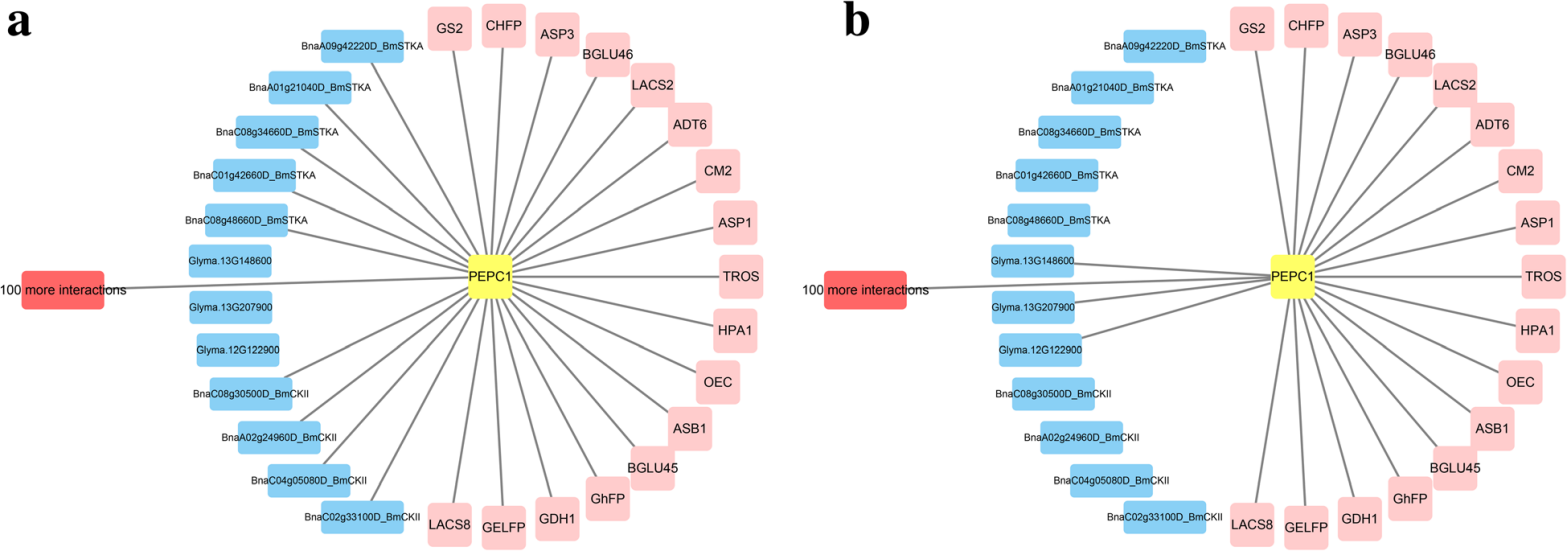
Comparison of *PEPC1* gene co-expression networks in rapeseed (**a**) and soybean (**b**). 17 genes in light pink are orthologous genes in rapeseed and soybean. More than 100 genes in red were enriched in the same processes based on KEGG pathway enrichment analysis. The blue nodes represent rapeseed genes enriched-specific in “plant circadian rhythms” (**a**) and soybean genes enriched-specific in “Valine, leucine and isoleucine biosynthesis” (**b**), respectively

The co-expression network of one gene is frequently constructed by Pearson’s correlation coefficient [65, 66]. In the present study this method was used to construct the co-expression networks of the *PEPC1* gene in soybean and rapeseed. The differences between the two networks were also used to identify extra candidate genes. As a result, 121 soybean and 133 rapeseed genes were co-expressed with *GmPEPC1* and *BnPEPC1*, respectively. Among these co-expressed genes, 17 were orthologous. The other genes were used to conduct KEGG pathway enrichment analysis. In the top 10 KEGG pathways for soybean or rapeseed, the soybean-specific biological process is “the synthesis of valine, leucine and isoleucine”, involving *Glyma.13G148600, Glyma.13G207900* and *Glyma.12G122900*, and rapeseed-specific biological process is “plant circadian rhythms”, involving genes *BnSTKA* (*BnaC08g48660D, BnaA09g42220D, BnaA01g21040D, BnaC08g34660D, BnaC01g42660D*) and *BnCKII* (*BnaC08g30500D, BnaC02g33100D, BnaC04g05080D, BnaA02g24960D*) (Fig. 3).

## Discussion

### PEPC, along with its miRNA and co-expressed genes, which can affect the flow of carbon sources in seeds, may contribute to the difference of seed oil content between soybean and rapeseed

**Fig. 4:**
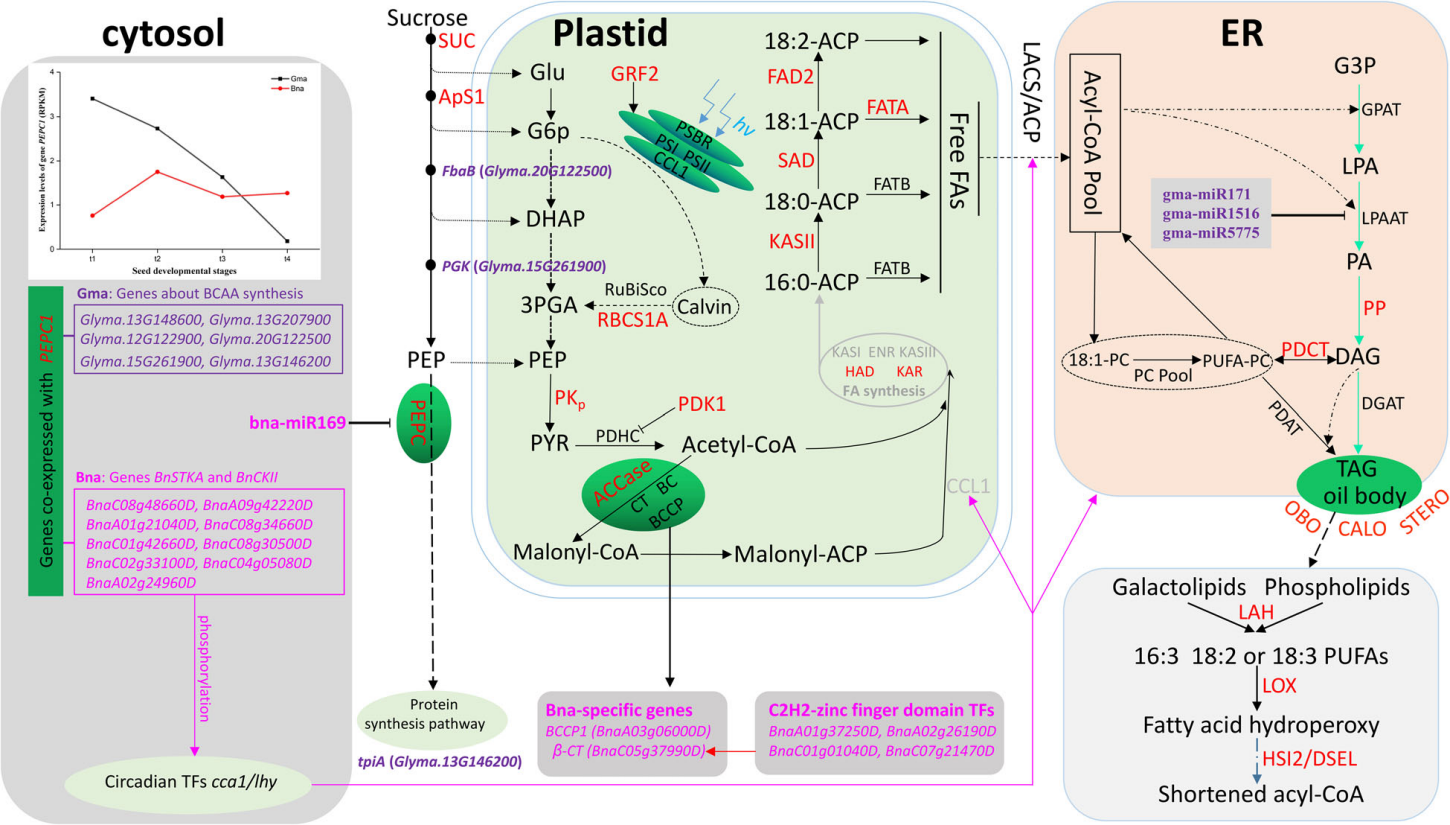
Molecular mechanisms for the difference of seed oil content between soybean and rapeseed. Candidate genes contributed to the differences of seed oil content between soybean and rapeseed obtained in the study were marked with red color. *GRF2* and *RBCS1A*: photosynthesis; *PGK*, *ApS1*, *SUC*, *PEPC* and *PKp*: carbon metabolism from sucrose to pyruvate; *PDK1*, *ACCase*, *KASII*, *HAD*, *KAR*, *FATA*, *SAD* and *FAD2*: in de novo fatty acid biosynthesis; *PAP* and *PDCT*: TAG synthesis; *OBO*, *CALO* and *STERO*: oil-body protein genes; *LOX*, *LAH*, *HSI2* and *DSEL*: oil degradation genes. Among these candidate genes, *BCCP1* (*BnaA03g06000D*) and *β-CT* (*BnaC05g37990D*) in heterogeneous acetyl-CoA carboxylase (ACCase) are rapeseed-specific genes, and *β-CT* is positively regulated by four transcription factors (*BnaA01g37250D, BnaA02g26190D, BnaC01g01040D* and *BnaC07g21470D*). The gene expression of *PEPC1* in rapeseed is putatively inhibited by bna-miR169, while *LPAAT* in soybean putatively inhibited by gma-miR171, gma-miR1516 and gma-miR5775 in triglyceride synthesis. The pink genes are speculated related specifically to high seed oil content of rapeseeds, and the purple speculated specifically related to high seed protein content in soybean, which were both identified by *PEPC* co-expression network analysis. Soybean genes participated in Branched-Chain Amino Acid (BCAA) synthesis may contribute to seed high protein content by adjusting the flow of PEP and downstream protein biosynthesis. Rapeseed genes *BnSTKA* and *BnCKII* are likely to promote the triglyceride synthesis by phosphorylating circadian TFs cca1/lhy and thus increase the seed oil content of rapeseed

Seed oil content is almost negatively correlated to seed protein content in soybean and rapeseed [67–69]. As we know, PEP is used to synthesize acetyl-CoA under the catalysis of pyruvate kinase (PK) and acetyl coenzyme A carboxylase (ACCase) so that the PEP enters into the fatty acid synthesis pathway. Additionally, PEP is also used to synthesize oxaloacetate (OAA) under the catalysis of phosphoenolpyruvate carboxylase (PEPC) so that the PEP enters into the amino acid synthesis pathway. Results in this study showed that PEPC genes, together with their miRNA and co-expressed genes, may increase the flow of carbon to the biosynthesis of amino acids in soybean seed and to the de novo fatty acid synthesis in rapeseed seed, resulting in the difference of seed oil content between the two species (Fig. 4). The reasons are as follows.

First, *GmPEPC1* has higher relative expression at the early and middle stages of seed development than *BnPEPC1*, and bna-miR169 putatively inhibits the expression of *BnPEPC*. Although the bacterial-type PEPC (BTPC) gene in *A. thaliana* can inhibit the expression of the plant-type PEPC (PTPC) gene [63, 70–73], BTPC genes in soybean have experienced positive selection (Additional file 3: Tables S7 and S8) and it is possible to lose the function of inhibiting the expression of PTPC gene (*GmPEPC1*). In addition, Xu et al. [12] increased the accumulation of cotton seed oil by the down-regulation of *GhPEPC1* via RNA interference in transgenic cotton plants. These studies provide evidence for greater carbon flow to amino acid metabolism in soybean seed and to de novo fatty acid synthesis in rapeseed seed. This may partly explain why there are high seed protein content in soybean and high seed oil content in rapeseed.

Secondly, the expression of LPAAT2-encoding gene, involved in triacylglycerol synthesis in soybean seed, is putatively inhibited by three miRNAs (gma-miR171, gma-miR1516 and gma-miR5775) based on the results of bioinformatics analysis (Table 2).

Finally, gene co-expression network analysis helps us to understand different biological pathways in soybean and rapeseed. KEGG enrichment analyses of genes co-expressed with *GmPEPC1* showed that three soybean genes (*Glyma.13G148600, Glyma.13G207900* and *Glyma.12G122900*) were enrich-specific in the “leucine, isoleucine and valine” synthesis pathway. Leucine, isoleucine and valine are the three major branched-chain amino acids for protein synthesis. The content of branched-chain amino acids in seeds is positively correlated with the protein content in general, which can effectively maintain the accumulation of storage proteins in seeds [74]. Therefore, it is speculated that the expression of such soybean genes may be beneficial for the accumulation of storage proteins in seeds. Meanwhile, KEGG enrichment analysis of genes co-expressed with *BnPEPC1* revealed that nine rapeseed genes, encoding serine/threonine-protein kinase (*BnSKTA*; *BnaC08g48660D, BnaA09g42220D, BnaA01g21040D, BnaC08g34660D* and *BnaC01g42660D*) and Casein kinase II subunit beta (*BnCKII*; *BnaC08g30500D, BnaC02g33100D, BnaC04g05080D* and *BnaA02g24960D*), were specifically enriched in the “plant circadian rhythm” category, which can regulate seed oil metabolism and hormone signaling pathway [75, 76]. Lipid metabolism is subject to diurnal regulation at the early stages of seed development in *Arabidopsis* [77]; diurnal differential expression of genes encoding JcDof1, a dof TF of *Jatropha curcas* in response to light signal, β-hydroxy-3-methylglutaryl-CoA reductase and Cyp7A1, regulates seed oil synthesis and accumulation [78, 79]. CIRCADIAN CLOCK ASSOCIATED1 (CCA1) and LATE ELONGATED HYPOCOTYL (LHY) from the core clock system can affect the reserve mobilization of storage lipid [77], but this process is affected by the phosphorylation of protein kinase (CK2) [80]. Therefore, the phosphorylation of genes *BnSTKA* and *BnCKII* may promote the storage of seed oil.

### Rapeseed-specific genes encoding β-CT and BCCP1 subunits of acetyl-CoA carboxylase and transcription factors may be associated with higher seed oil content in rapeseed

β-CT and BCCP are important components for heterogeneous acetyl-CoA carboxylase (ACCase) [81, 82]. Overexpression of ACCase subunit genes can significantly increase fatty acid content in oil crop seed [13, 14, 83]. In this study, soybean β-CT and BCCP1 was not expressed, while BCCP2, BC and α-CT showed high expression during seed development stages (Additional file 2: Figure S4). This is consistent with the results of Zhang et al. [55]. Meanwhile, genes BCCP1, BCCP2, BC, α-CT and β-CT showed high expression during rapeseed seed development stages. Especially, the β-CT subunit gene (*BnaA10g13960D*) was positively regulated by four zinc finger C2H2 transcription factors (*BnaA01g37250D, BnaA02g26190D, BnaC01g01040D* and *BnaC07g21470D*) predictably, which is consistent with the results of Jin et al. [62]. Similarly, Li et al. [36] demonstrated that overexpression of *GmZF351*, a gene encoding a tandem CCCH zinc finger protein, can activate lipid biosynthesis genes and increase seed oil accumulation in soybean. Moreover, Li et al. [84] found that transfer DNA insertional alleles that completely eliminate the accumulation of BCCP2 have no perceptible effect on fatty acid accumulation, while reducing the BCCP1 accumulation can dramatically decreases fatty acid accumulation in *Arabidopsis thaliana*. This partly supports that rapeseed-specific *BCCP1* gene may associate with high seed oil content of rapeseed. It should also note that RNA levels don’t always equate to protein and/or lipid metabolite levels in plants [85].

### The expansion of gene families associated with lipid storage and the contraction of gene families related to lipid degradation may contribute to high seed oil content in rapeseed

Seed triglyceride is mainly stored in lipid droplets, and the size of the lipid droplets and the spatial distribution of their assembly proteins affect seed oil content [86, 87]. In this study, it was found that the relative copy numbers of genes encoding STERO, CALO and OLEs in rapeseed are significantly higher than those in soybean (Fig. 1, Additional file 1: Table S6), and such genes in rapeseed are obviously up-regulated during stages of rapid lipid accumulation (t2~t3) (Additional file 2: Figure S3, Additional file 1: Table S6). On the other hand, the gene families LOX, LAH and HSI2, related to lipid degradation, have contracted in rapeseed. In other words, the relative copy numbers of these genes are much smaller than those in soybean (31/4 < 41/2, 10/4 < 24/2 and 8/4 < 8/2, (gene absolute copy numbers) / (species polyploidy)). This relationship was also found between soybean and sesame. Specifically, this latter species shows contraction of gene families (LOX, LAH and FAR1) related to lipid degradation and expansion of gene families (LTP1 and SUT) related to lipid storage [51]. Therefore, it was speculated that the contraction of gene families related to lipid degradation and the expansion of gene families related to lipid storage may be an important reason for the higher seed oil content in rapeseed than in soybean.

In order to further ascertain whether the degradation of seed storage materials in oilseed crop is specialized in an evolutionary sense, we investigated gene families related to protein degradation in soybean, rapeseed and sesame seeds. As we know, the degradation of protein in plant cells is mainly mediated by the ubiquitin proteasome, lysosomal and caspase pathways. Among the three pathways, the ubiquitination proteasome pathway is the main pathway of storage protein degradation in oilseed crop seeds [88], and it mainly involves ubiquitin-activating enzyme (E1), ubiquitin-conjugating enzyme (E2) and ubiquitin ligases (E3) [89]. In this study, we found that the relative copy numbers of genes encoding E1, E2 and E3 in soybean, rapeseed and sesame were 4/2 = 8/4 > 2/2, 44/2 < 105/4 > 23/2 and 33/2 > 52/4 > 10/2, which are not consistent with the protein contents in soybean, rapeseed and sesame seeds (~ 40%, ~ 20%, and ~ 17%). This indicates that the phenomenon, which has a bias to consume protein or oil mainly to power the life activities during seed development, does not occur in the evolution of oil crops.

### More evidence for candidate genes that are associated with the seed oil content difference between soybean and rapeseed

Many candidate genes predicted in this study could be responsible for the difference of seed oil content between soybean and rapeseed have been experimentally confirmed to be related to seed oil content. In addition to those mentioned above, with the up-regulated expression for the genes *BnGRF2* and *BnRBCS1A* and the down-regulated expression of gene *BnPDK1*, Hu et al. [69] cultivated a rapeseed line YN171 with a super high seed oil content of 64.8% (Fig. 1). Similarly, with the down-regulated expression of gene *GmFAD2–1* by RNA interference, seed oleic acid content in soybean increased to 94.58% and the linoleic acid content decreased to < 3% (Fig. 1) [15].

Differentially expressed genes, associated with seed oil content and identified among cultivars with different seed oil content, also provide relevant evidence. Among the 33 differentially expressed genes identified in rapeseed by Xu et al. [90], PDAT (Additional file 1: Table S4) and OBO (Fig. 1) are also found in the present study. Among the 28 core enzymes involved in lipid synthesis in soybean [55], 8 were also found in the present study (Additional file 3: Table S9).

In this study, we are focusing on the difference of total seed oil content between soybean and rapeseed. However, their other differences exist as well, i.e., seed oil composition, grown climatic environment, nitrogen fixation, and species characteristics, which likely affect the conclusion in this study.

## Conclusion

In this study, we identified candidate genes and their transcription factors and microRNAs to explain the difference in seed oil content between soybean and rapeseed. First, *PEPC*, along with its microRNAs and co-expression genes, affect the carbon source flow in seeds, which may lead to differences in seeds oil content. Then, *BCCP1* and *β-CT* and its transcription factors that are characteristic of rapeseed may result in high seeds oil content. Finally, the expansion of gene families related to lipid storage, and the contraction of gene families related to oil degradation may play important roles on the difference in seed oil content.

## Methods

### Data sources

Sequences were collected using the similar method described by Tatusov et al. [91]. Protein-coding transcripts of *Arabidopsis* (TAIR release 10, https://www.arabidopsis.org/), rapeseed (release 4.1, http://www.genoscope.cns.fr/brassicanapus/), soybean (release Wm82.a2.v1, https://www.soybase.org/) and sesame (release 1.0, http://ocri-genomics.org/Sinbase/) were downloaded, respectively. If a gene has multiple transcripts, the longest was selected.

The transcriptome data of soybean (*G. max* Williams 82) [58] (https://www.ncbi.nlm.nih.gov/geo/query/acc.cgi?acc=GSE42871) and rapeseed (*B. napus* Darmor-*bzh*) [57] (https://www.ncbi.nlm.nih.gov/geo/query/acc.cgi?acc=GSE77637. ) were downloaded from Gene Expression Omnibus (GEO). Rapeseed transcriptome data included four seed developmental stages: 2, 4, 6, and 8 weeks after pollination (WAP), and soybean transcriptome data included seven seed developmental stages: whole seed 4 days after fertilization (DAF), whole seed 12–14 DAF, whole seed 22–24 DAF, whole seed 5–6 mg in weight, cotyledons 100–200 mg in weight, cotyledon 400–500 mg in weight, and dry whole seed. Of which, whole seed 12–14 DAF, whole seed 22–24 DAF, cotyledon 400–500 mg in weight, and dry whole seed in soybean are almost respectively equal to 2, 4, 6, and 8 DAF based on the definition of soybean vegetative and reproductive growth [92], which are consistent with 2, 4, 6, and 8 WAP in rapeseed. Thus, we selected the four stages of soybean and rapeseed seed development mentioned above for subsequent analysis. The genes expression level (RPKM: reads per kilobase per million mapped reads) were normalized and quantified by the DESeq package in Bioconductor [93].

### Delimitation of orthologous genes

Identification of orthologous groups (OGs) in *Arabidopsis*, soybean, rapeseed and sesame was conducted using OrthoMCL software with default parameters [94]. Based on all-against-all BLASTP of non-redundant protein sequences, clusters were obtained according to reciprocal best similarity pairs between and within species, using OrthoMCL software implemented by the Markov clustering algorithm (MCL; http://micans.org/mcl/) [95]. To obtain more accurate results, two other known methods, namely Proteinortho [96] and Inparanoid 8 [97], were also used to determinate OGs of soybean and rapeseed.

### Prediction of candidate genes related to carbon metabolism and lipid biosynthesis

Acyl-lipid biosynthesis process is mainly involved in fatty acid synthesis and elongation from pyruvate, TAG synthesis, and oil-body storage. In *Arabidopsis*, 135 acyl-lipid biosynthesis-related genes were downloaded from ARALIP (http://aralip.plantbiology.msu.edu/) [9], and 238 carbon metabolism-related genes were also obtained using the method of Troncoso-Ponce et al. [10] with a slight modification. Such genes were used as query, along with OGs, to identify carbon metabolism- and lipid biosynthesis-related OGs. To further identify candidate genes for the differences of seed oil content, gene expression pattern clustering and interspecific relative copy numbers variation analysis were carried out. Gene expression clustering analysis was performed using Short Time-series Expression Miner (STEM, http://www.cs.cmu.edu/~jernst/stem/) [56] with the following parameters: Log normalize a time series vector of gene expression values (v_0_, v_1_, v_2_, ..., v_*n*_) to (0, log_2_(v_1_/v_0_), log_2_(v_2_/v_0_), ⋯, log_2_(v_*n*_/v_0_)), Minimum Absolute Expression Change 2, −p 0.05.

### KEGG pathway enrichment analysis

Kyoto Encyclopedia of Gene and Genome (KEGG) pathway enrichment analysis was performed using the online tool KOBAS (version 2.0; http://kobas.cbi.pku.edu.cn/index.php) [59]. The *P*-values for each KEGG biological process was calculated by Fisher’s exact test [59]. To control the false discovery rate (FDR ≤ 0.05), the Benjamini-Hochberg method was used to conduct multiple testing correction [98]. In addition, the small term cutoff value was set at 5.

### Phylogenetic analysis and motif analysis

The full-length amino acid sequence alignments were performed using MUSCLE [99] with default parameters and then phylogenetic tree reconstruction was conducted with both Neighbor Joining (NJ) and Maximum Likelihood (ML) approaches in MEGA 7.0 [100]. In the NJ method, parameter setups were as follows: - model: poisson correction; Bootstrap: 1000 replicates; and gap/missing data: pairwise deletion. To ensure the accurateness of ML tree, which is constructed to eliminate the long-branch attraction (LBA) caused by distant species, we also used maximum likelihood approaches with PhyML v3.0 [101], and estimated the best-fitting models with the jModeltest software [102]. The phylogenetic tree was displayed, annotated and managed using iTOL (https://itol.embl.de/) [103]. Conserved functional motifs were identified using the program Multiple Em for Motif Elicitation [64] (MEME v4.11.2, http://meme-suite.org/tools/meme) with the following parameters: - the width of a motif was between 6aa and 50aa, and the number of motifs was no more than 20.

### Selective pressure and positive selection analyses

The amino acid sequences were aligned using MUSCLE [99], alignment gaps were manually deleted, and then used for following calculations. The ratio (ω value) of nonsynonymous substitution rate (Ka) to synonymous substitution rate (Ks) of homologous gene pairs was computed with the maximum likelihood method of the branch model in Codeml from the PAML package (version 4.9) [104].

To test for the variation of the ω ratio among different branches in gene trees, a branch-specific model was used and conducted in Codeml. The branch-specific model allows the ω ratio to vary among branches in the phylogeny (model = 2, NSsites = 0), and it could be used to test whether there are different ω values on particular lineages [105]; thus, this model can be compared with the one-ratio model (model = 0, NSsites = 0) that assumes a constant ω value across all branches using the likelihood ratio test (LRT). The datasets used in Ka/Ks ratio estimation were further used in the next positive selection analysis of the branch-site model (BSM) using the Bayes empirical Bayes method described by Yang et al. [104].

### Transcription factor (TF)- and microRNA-targets analysis

Soybean and rapeseed microRNAs were downloaded from miRBase (release 21, http://www.mirbase.org/) [106]. psRNATarget (http://plantgrn.noble.org/psRNATarget) [107] was used to identify miRNA targets with default parameters except for the Expectation (e) and Max UPE, which were set at 3 and 25, respectively. The transcription factors (TFs) and TF-target pairs were downloaded directly from PlantTFDB 3.0 (http://planttfdb.cbi.pku.edu.cn/) [61]. To ascertain whether miRNAs controls target-genes expression in seed development stages, bioinformatic analysis software miRDB (http://mirdb.org/miRDB/) [108] was used to preliminarily verify whether there is a putative binding site for miRNAs in the 3′-UTR of target-genes mRNA.

### Gene co-expression network analysis

Pearson’s correlation coefficients (*r*) were calculated using the ‘cor’ function of R package. The gene expression data (Reads Per Kilobase per Million mapped reads: RPKM) was used to calculate the correlation coefficients between genes. The criteria for determining co-expressional genes were set at *r* ≥ 0.9 or *r* ≤ − 0.9 and *P*-values ≤ 0.05 [65]. Graphical visualization of the gene co-expression network was performed using Cytoscape 3.4.0 (http://www.cytoscape.org/) [109]. Genes co-expressed with the target gene, meeting the filter criteria, were further used to conduct KEGG pathway enrichment analysis using KOBAS 2.0 [59].

## Additional files

Additional file 1: **Table S1.** 27,236 OGs of all the protein-coding genes in *Arabidopsis thaliana, Glycine max, Brassica napus* and *Sesamum indicum*. **Table S2.** Comparisons of sequence similarity-based protein families between *Glycine max* (gma) and *Brassica napus* (bna). **Table S3.** List of selected genes related to carbohydrate metabolism and lipid biosynthesis in *Arabidopsis thaliana*. **Table S4.** 230 candidate orthologous groups related to oil synthesis of seed for soybean, rapeseed and *Arabidopsis*. **Table S5.** Candidate orthologous groups (OGs) for the difference of seed oil content between rapeseed and soybean. Note: Genes with red color were negatively correlated with seed oil content and were obtained by cluster analysis of gene expression. The 44 OGs with black color differed in gene relative copy number between soybean and rapeseed. **Table S6.** Expressional contents and relative copy number of candidate genes associated with the difference of seed oil content in rapeseed and soybean. Note: The stages t1, t2, t3 and t4 were defined as R3, R4, R7 and R8 in soybean, and 2, 4, 6, and 8 weeks after pollination (WAP) in rapeseed, respectively. (XLSX 37341 kb)

Additional file 2: **Figure S1.** The expression patterns for the genes of 230 gene families related to oil biosynthesis. The expression clustering analysis of 2048 soybean and rapeseed genes in the 230 gene families was performed using Short Time-series Expression Miner (STEM, http://www.cs.cmu.edu/~jernst/stem/) [56]. Here, t1 represents the seed oil initial synthesis stage; t2 to t3 represent the rapid accumulation period of seed oil biosynthesis; t4 represents the gradual decline stage after the seed oil accumulation content reaches the peak. In the end, all 2048 genes were clustered into 20 clusters. **Figure S2.** The expression profiles (A-D) of candidate genes related to oil biosynthesis. One down-regulated trend (profile 3) (A) and three up-regulated trends from t2 to t3 stages of seed oil biosynthesis (profile 13, 16 and 18, respectively) (B, C, D). **Figure S3.** Comparison of the expression patterns of the candidate genes between rapeseed and soybean. Note: t1-t4 and t1’-t4’ represent four seed development stages in rapeseed and soybean, respectively. PKp-α and PKp-β denote Alpha (α) and Beta (β) subunits of PK in plastid, respectively. ACCase contains homogeneous structure ACC2 and heterogeneous ACCase complex, which are composed of α-CT, β-CT, BC and BCCP. **Figure S4.** Comparison of the expression patterns of genes encoding enzymes PEPC, PK and ACCase. t1’, t2’, t3’ and t4’ represent R3, R4, R7 and R8 at soybean seed development stages, and t1, t2, t3 and t4 represent 2, 4, 6 and 8 weeks after pollination (WAP) at rapeseed seed development stages, respectively. **Figure S5.** Transcriptional regulation of key candidate genes for the difference of seed oil content between rapeseed and soybean. **Figure S6.** Evolutionary rate of each branch of PEPC gene family. ω0 = 0.340 represents the evolutionary rate when the evolutionary rate of each branch is assumed to be the same. (PDF 1291 kb)

Additional file 3: **Table S7.** LRT results for selective pressure branch model (Model 0 vs two ratio model 2, *df* = 6). **Table S8.** LRT results for branch-site model (model A vs null model, *df* = 1). **Table S9.** Candidate genes for the differences of seed oil content between the two species and among cultivars in the same species. (PDF 93 kb)

## Abbreviations

ACCase: Acetyl CoA carboxylase
ApS1: ADP-glucose pyrophosphorylase
CALO: Caleosin
DSEL: Phospholipase
FAD: Oleoyl desaturase
FATA: Fatty acylthioesterase A
GEO: Gene Expression Omnibus
GRF2: Growth-regulating factor 2
HAD: Hydroxyacyl-ACP Dehydrase
HSI2: High-level expression of sugar-inducible gene 2
KAR: Ketoacyl-ACP reductase
KASII: Ketoacyl-ACP Synthase II
LAH: Lipid Acylhydrolase-like
LBA: Long-branch attraction
LOX: Lipoxygenase
LPAAT: Lysophosphatidic acid acyltransferase
MCL: Markov clustering algorithm
OAA: Oxalacetic acid
OBO: Oil-Body Oleosin
OGs: Orthologous groups
PAP: Phosphatidate phosphatase
PDCT: Phosphatidylcholine:diacylglycerol cholinephosphotransferase
PDK1: Pyruvate dehydrogenase kinase isozyme 1
PEP: Phosphoenolpyruvate
PEPC: Phosphoenolpyruvate carboxylase
PGK: Phosphoglycerate kinase
PK: Pyruvate kinase
PKp: Plastidial pyruvate kinase
PYR: Pyruvic acid
RBCS1A: Ribulose bisphosphate carboxylase small chain 1A
SAD: Stearyl-ACPdesaturase
STERO: Steroleosin
SUC: Sucrose transport protein
WAP: Weeks after pollination
