# Supplementary material for: An integrated omics analysis reveals molecular mechanisms that are associated with differences in seed oil content between Glycine max and Brassica napus
Source: BMC Plant Biol. 2018 Dec 4;18:328. doi: 10.1186/s12870-018-1542-8 (PMC6280547; doi:10.1186/s12870-018-1542-8)
Supplement: Supplementary file 2 — Figure S1. The expression patterns for the genes of 230 gene families related to oil biosynthesis. The expression clustering analysis of 2048 soybean and rapeseed genes in the 230 gene families was performed using Short Time-series Expression Miner (STEM, http://www.cs.cmu.edu/~jernst/stem/) [56]. Here, t1 represents the seed oil initial synthesis stage; t2 to t3 represent the rapid accumulation period of seed oil biosynthesis; t4 represents the gradual decline stage after the seed oil accumulation content reaches the peak. In the end, all 2048 genes were clustered into 20 clusters. Figure S2. The expression profiles (A-D) of candidate genes related to oil biosynthesis. One down-regulated trend (profile 3) (A) and three up-regulated trends from t2 to t3 stages of seed oil biosynthesis (profile 13, 16 and 18, respectively) (B, C, D). Figure S3. Comparison of the expression patterns of the candidate genes between rapeseed and soybean. Note: t1-t4 and t1’-t4’ represent four seed development stages in rapeseed and soybean, respectively. PKp-α and PKp-β denote Alpha (α) and Beta (β) subunits of PK in plastid, respectively. ACCase contains homogeneous structure ACC2 and heterogeneous ACCase complex, which are composed of α-CT, β-CT, BC and BCCP. Figure S4. Comparison of the expression patterns of genes encoding enzymes PEPC, PK and ACCase. t1’, t2’, t3’ and t4’ represent R3, R4, R7 and R8 at soybean seed development stages, and t1, t2, t3 and t4 represent 2, 4, 6 and 8 weeks after pollination (WAP) at rapeseed seed development stages, respectively. Figure S5. Transcriptional regulation of key candidate genes for the difference of seed oil content between rapeseed and soybean. Figure S6. Evolutionary rate of each branch of PEPC gene family. ω0 = 0.340 represents the evolutionary rate when the evolutionary rate of each branch is assumed to be the same. (PDF 1291 kb) [file 12870_2018_1542_MOESM2_ESM.pdf]

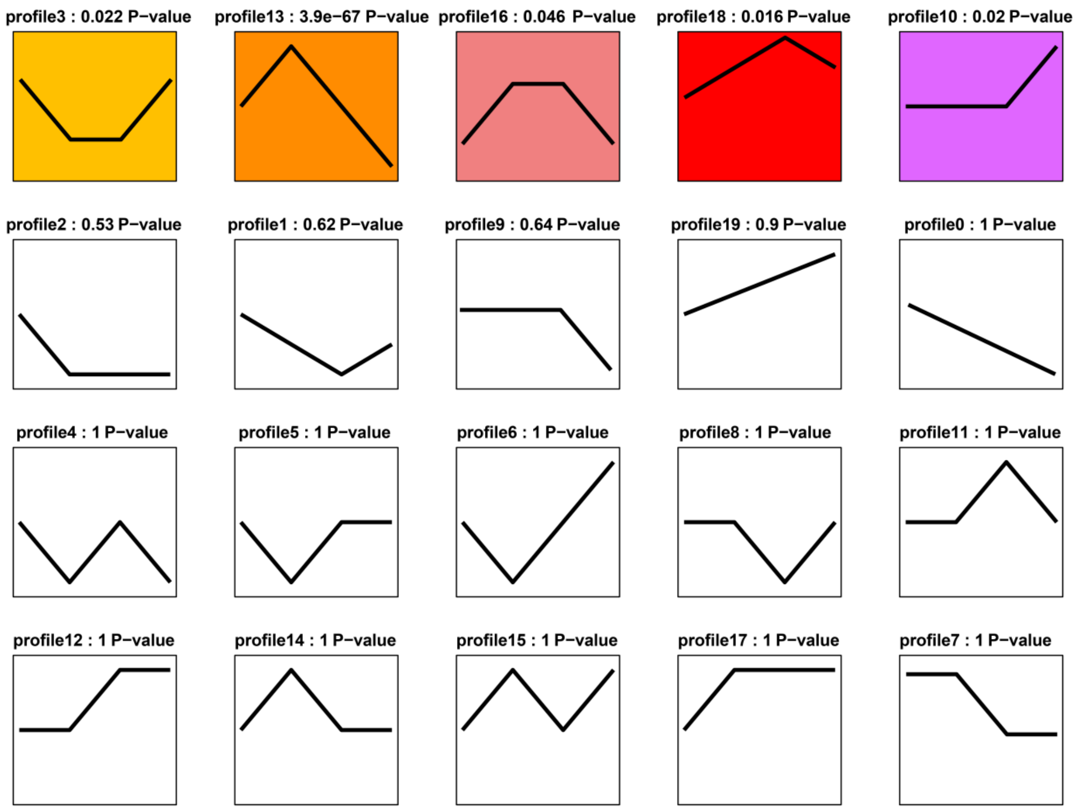

Figure S1. The expression patterns for the genes of 230 gene families related to oil biosynthesis. The expression clustering analysis of 2048 soybean and rapeseed genes in the 230 gene families was performed using Short Time-series Expression Miner (STEM, http://www.cs.cmu.edu/~jernst/stem/) (Ernst et al., 2006) with the following parameters: Log normalize a time series vector of gene expression values (v0, v1, v2, ..., vn) to (0, , ,), Minimum Absolute Expression Change 2, -p 0.05. In this study, t1 represents the seed oil initial synthesis stage; t2 to t3 represent the rapid accumulation period of seed oil biosynthesis; t4 represents the gradual decline stage after the seed oil accumulation content reaches the peak. In the end, all 2048 genes were clustered into 20 clusters.

Reference

1. Ernst J, Bar-Joseph Z. STEM: a tool for the analysis of short time series gene expression data. BMC Bioinformatics. 2006;7:191.

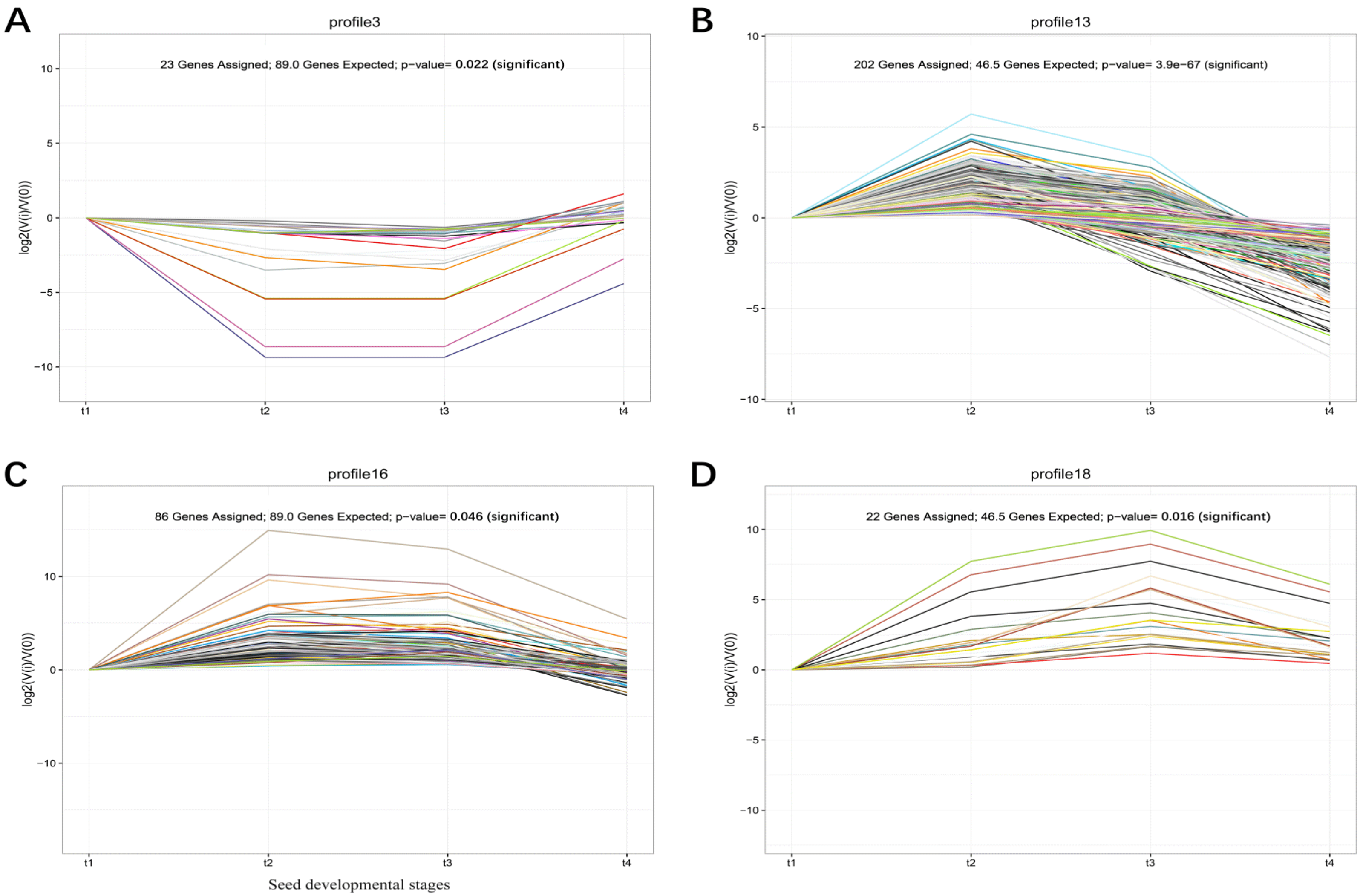

Figure S2. The expression profiles (A-D) of candidate genes related to oil biosynthesis. A down-regulated trend (profile 3) (A) and three up-regulated trends from t2 to t3 stages of seed oil biosynthesis (profiles 13, 16 and 18, respectively) (B, C, D).

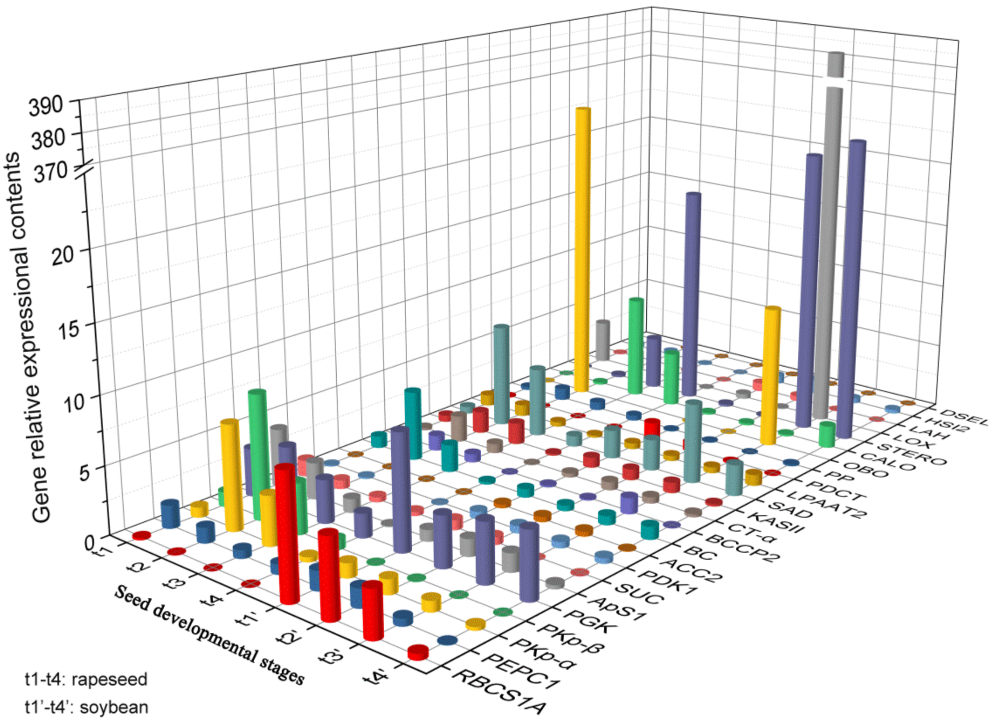

Figure S3. Comparison of the expression patterns of the candidate genes between rapeseed and soybean. t1-t4 and t1'-t4' represent four seed development stages in rapeseed and soybean, respectively. PKp-α and PKp-β denote Alpha (α) and Beta (β) subunits of PK in plastid, respectively. ACCase contains homogeneous structure ACC2 and heterogeneous ACCase complex, which are composed of α-CT, β-CT, BC and BCCP.

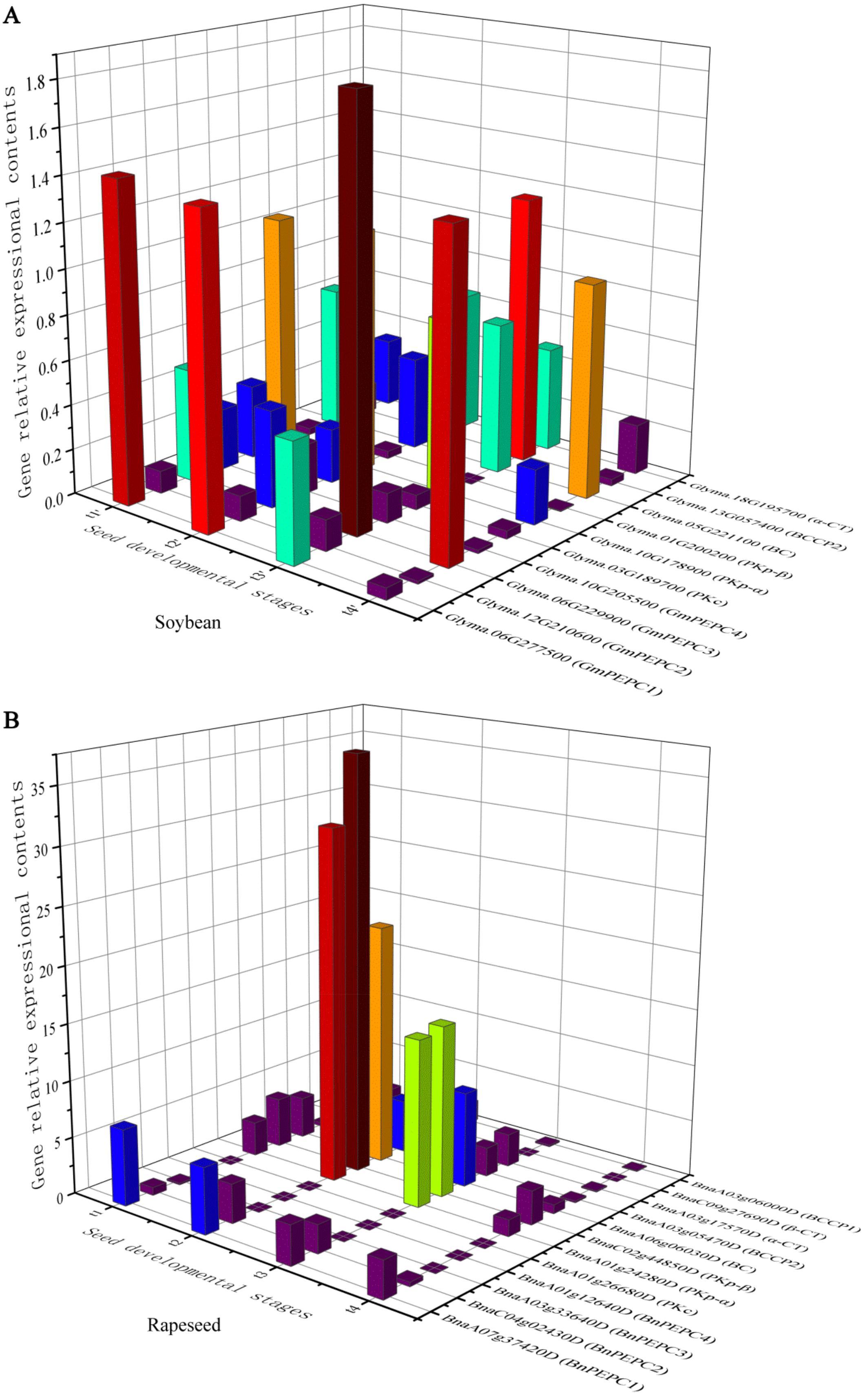

Figure S4. Comparison of the expression patterns of genes encoding enzymes PEPC, PK and ACCase. t1', t2', t3' and t4' represent R3, R4, R7 and R8 at soybean seed development stages **(A)**, and t1, t2, t3 and t4 represent 2, 4, 6 and 8 weeks after pollination (WAP) at rapeseed seed development stages **(B)**,

respectively. The expression levels of α-carboxyltransferase (a-CT), β-carboxyltransferase (β-CT), biotin carboxylase (BC) and biotin carboxylase (BCCP) of heterozygous ACCase were significantly higher than those of the corresponding homologous genes in soybean in the seed development stages. The expression of BCCP1 and BCCP2, two subtypes of BCCP, were both detected in rapeseed, while only the expression of BCCP2 was detected in soybean. β-CT was not found to be expressed during soybean seed development stages and GmPEPC1 (Glyma.06G277500) and GmPEPC3 (Glyma.06G229900) had higher relative expression than PKp and ACCase in soybean. Conversely, PKp-β (BnaC02g44850D), PKp-α (BnaA01g24280D) and ACCase had higher relative expression than PEPC in rapeseed. Another type of acetyl coenzyme A carboxylase (homogeneous ACC2) was detected in both rapeseed and soybean seeds, and their expression levels were almost relatively same conservative trend.

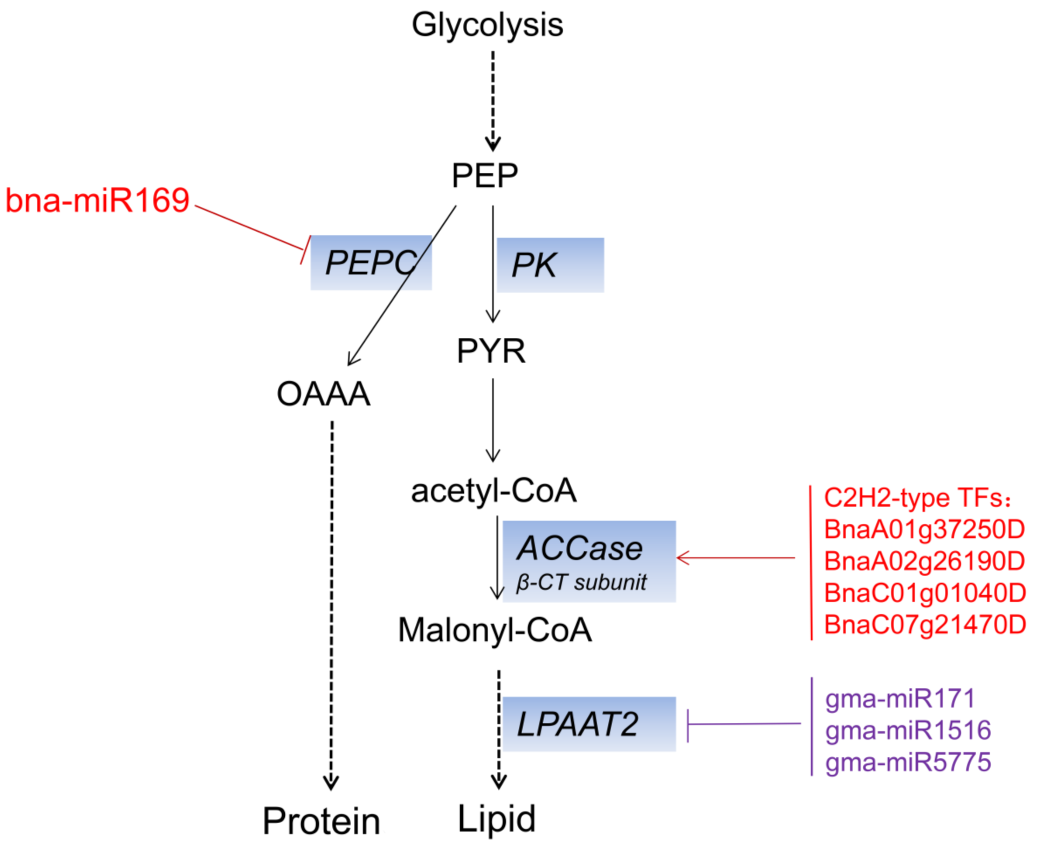

Figure S5. Transcriptional regulation of key candidate genes for the difference of seed oil content between rapeseed and soybean. In rapeseed, the bna-miR169 putatively inhibits the expression of gene BnPEPC, and the expression of β-CT subunit of ACCase is putatively regulated by four zinc finger family transcription factors, while the expression of GmLPAAT2 gene in soybean is putatively inhibited by gma-miR171, gma-miR1516 and gma-miR5775.

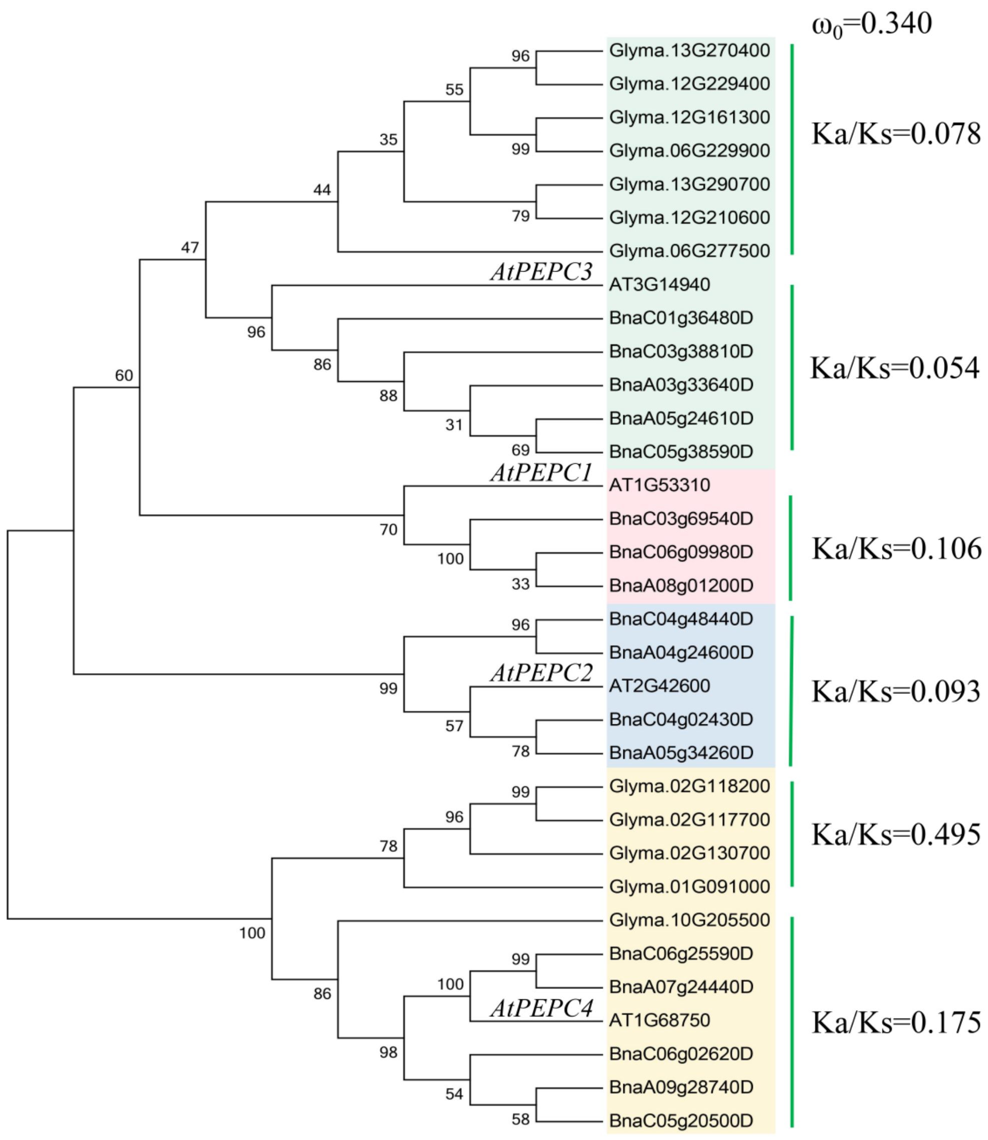

Figure S6. Evolutionary rate of each branch of *PEPC* gene family. ω_0=0.340 represents the evolutionary rate when the evolutionary rate of each branch is assumed to be the same.
