# Supplementary material for: An integrated omics analysis reveals molecular mechanisms that are associated with differences in seed oil content between Glycine max and Brassica napus
Source: BMC Plant Biol. 2018 Dec 4;18:328. doi: 10.1186/s12870-018-1542-8 (PMC6280547; doi:10.1186/s12870-018-1542-8)
Supplement: Supplementary file 3 — Table S7. LRT results for selective pressure branch model (Model 0 vs two ratio model 2, df = 6). Table S8. LRT results for branch-site model (model A vs null model, df = 1). Table S9. Candidate genes for the differences of seed oil content between the two species and among cultivars in the same species. (PDF 93 kb) [file 12870_2018_1542_MOESM3_ESM.pdf]

Table S7. LRT results for selective pressure branch model (Model 0 vs Two ratio Model 2, df=6)

Model	np	LnL	Model compared	LRT P-value
Model 0	71	-2343.655		
Model 2	65	-2361.163	Model 0 VS Two ratio Model	P-value=4.278e-06**

Note: ** significant at the 0.01 level.

Table S8. LRT results for branch-site model (Model A vs Model null, df =1)

Model	np	LnL	Estimates of parameters					Model compared	LRT P-value	Positive sites
Model A	68	-2328.087976	Site class	0	1	2a	2b			
			f	0.86630	0.06859	0.06033	0.00478	Model A vs Model A null	0.001684**	56F 0.998** 61V 0.950*
			ω0	0.08364	1.00000	0.08364	1.00000			
			ω1	0.08364	1.00000	999.00000	999.00000			
Model A null	67	-2333.020693	1							

Note: The analysis was performed by the EasyCodeML_v1.0.

Table S9. Candidate genes related to differences in seed oil content

	Candidate genes related to the differences of interspecific seed oil contents	Lipid-related DEGs between varieties	
		Among three *Glycine max* varieties	Among two *Brassica napus* varieties
Gene ID / proteins Abbreviation	*GRF2, RBCS1A*<br>*PGK, APS1*<br>*SUC, PEPC*<br>*PKp, PDK1*<br>*ACC2, ACCase HAD, KASII*<br>*KAR, FATA*<br>*SAD, FAD2 PAP, PDCT*<br>*OBO, CALO*<br>*STERO, LOX*<br>*LAH, HSI2, DSEL*	*Glyma08g47240_ABI3*<br>*Glyma07g05550_α-PDH*<br>*Glyma19g03530_BCCP2*<br>*Glyma02g08600_CK*<br>*Glyma09g07520_DGAT1*<br>*Glyma03g30070_FAD2*<br>*Glyma15g05800_HAD*<br>*Glyma17g05200_KASII*<br>*Glyma13g11700_LACS*<br>*Glyma04g08220_OLE*<br>*Glyma13g16790_PDAT1*<br>*Glyma04g04060_PAP*<br>*Glyma14g27920_SAD*<br>*Glyma01g43780_STERO*<br>*Glyma11g31234_VAL*<br>*Glyma12g13300_GmTZF4*<br>*Glyma07g04290_GmGRF5*<br>*Glyma10g08370_ABI5*<br>*Glyma03g34730_Trihelix*	*chrC06-6493884-6495189_At3g13900*<br>*chrC08-38275473-38277304_PI5P-II*<br>*chrAnn_random-16760656-16761926_GPI-PLC*<br>*chrA03-6423275-6428210_MCMT*<br>*chrA10_random-2008292-2010065_PDAT*<br>*chrC05-41766918-41768242_MFP*<br>*chrA03-17293983-17296995_OBO*<br>*chrC08-8727719-8730221_Sac-PIP*
References		Zhang et al. (2016)	Xu et al. (2015)

References

1. Xu HM, Kong XD, Chen F, Huang JX, Lou XY, Zhao JY. Transcriptome analysis of *Brassica napus* pod using RNA-seq and identification of lipid-related candidate genes. BMC Genomics. 2015;16:858.
2. Zhang L, Wang SB, Li QG, Song J, Hao YQ, Zhou L, Zheng HQ, Dunwell JM, Zhang YM. An integrated bioinformatics analysis reveals divergent evolutionary pattern of oil biosynthesis in high- and low-oil plants. PLoS One. 2016;11(5):e0154882.
